# Incidence and Causes of Stillbirth in Omdurman Maternity Hospital, Sudan: A Prospective Cross-Sectional Study

**DOI:** 10.7759/cureus.15453

**Published:** 2021-06-05

**Authors:** Mohamed Alkhatim Alsammani

**Affiliations:** 1 Department of Obstetrics and Gynaecology, University of Bahri, Khartoum, SDN; 2 Department of Obstetrics and Gynecology, College of Medicine, Qassim University, Buraidah, SAU

**Keywords:** stillbirth, causes, incidence, risk, sudan

## Abstract

Background: Stillbirth is an important indicator of the quality of antenatal health services. This study aimed to identify the incidence and causes of stillbirths among Sudanese women.

Method: This is a descriptive cross-sectional hospital-based study that was conducted at Omdurman Maternity Hospital during the period from December 1, 2019 to May 30, 2020. The study sample comprised 285 women who presented with stillbirths. Data were collected using a structured questionnaire administered to women after taking informed consent. Data were analyzed using descriptive statistics [Statistical Package for Social Sciences (SPSS) version 24 (IBM Corp., Armonk, NY)].

Results: The incidence of stillbirths was 16/1000. Idiopathic causes were the most frequent causes which accounted for 20% (n=57), followed by pre-eclampsia 18.6% (n=53), congenital abnormalities 15.1% (n=43), and abruption placentae 14.4% (n=41). In addition, the less common causes were eclampsia 4.6% (n=13), ruptured uterus 4.2% (n=12), twin-twin transfusion 4.2% (n=12), cord prolapse 3.5% (n=10), uncontrolled diabetes mellitus (DM) 3.5% (n=10), malpresentation 2.6% (n=8), gestational DM 2.5%(n=7), anemia 2.5%(n=7), sepsis 2.1 (n=6), placenta previa 1.4% (n=4), renal disease 0.4% (n=1), and toxoplasmosis 0.4% (n=1).

Conclusion: The incidence of stillbirths was 16/1000. Unexplained causes of stillbirths were the most common causes which accounted for 20% of all deaths. In contrast, explainable causes were responsible for 80% of fetal deaths. Among explainable causes, pre-eclampsia and its consequences (abruption, eclampsia) remain the most common cause.

## Introduction

The Perinatal Mortality Surveillance Report Stillbirth describes the death of a baby in the uterus, and the term stillbirth applies to a baby delivered with no signs of life [1]. 

The global incidence of stillbirths ranges from 3.4/1000 total births in high-income countries to as high as 36/1000 in the Sub-Saharan and Asia regions [2]. The incidence in the UK ranges between 33.9/1000 in North Ireland and 4.6/1000 in Scotland [1].

Factors commonly associated with stillbirths include nulliparity and grand multiparity, obesity, uncontrolled diabetes mellitus (DM), antepartum bleeding, and advanced maternal age, especially over 40 years of age [2-3]. However, the cause of death in the majority of cases remains unexplained [3]. 

Intrapartum causes of stillbirths include fetal birth asphyxia, birth trauma, lung immaturity, intracranial hemorrhage, and neonatal infection [3-4]. It has been reported that the causes of perinatal mortality change due to the progress in prenatal diagnosis and perinatal management [5-6]. Fresh stillbirth reflects the quality of intrapartum care, while macerated stillbirth reflects antenatal care (ANC) quality [7-8].

There was a huge positive change in ANC over the past decades. Evidence indicates that early visits positively impact maternal and fetal health due to early diagnosis, intervention, and screening programs [9]. However, studies’ results have shown that fetal deaths can occur even in women who had adequate ANC [10].

Literature on stillbirths is scant and not updated among Sudanese pregnant women. Therefore, the present study aimed to determine the incidence and causes of stillbirth deaths among pregnant women attending Omdurman Maternity Hospital.

## Materials and methods

Study design and setting

This is a descriptive cross-sectional hospital-based study conducted at Omdurman Maternity Hospital from December 1, 2019 to May 30, 2020. Omdurman Maternity Teaching Hospital was found in 1957 and it is the largest tertiary center that provides all obstetrical services at a tertiary level.

Diagnosis

The diagnosis of stillbirths was based on the Royal College of Obstetricians and Gynaecologists' (RCOG) recommendations. Real-time ultrasound was used in suspected stillbirths and the diagnosis was established when demonstrating the absence of fetal cardiac activity, with or without secondary features: spalding sign, fetal hydrops, and presence of air in great vessels [1].

Definitions

*Stillbirth*: stillbirth is defined as a baby delivered dead with no signs of life after 24 completed weeks of pregnancy and with no signs of life in utero [1].

*Unexplained stillbirth*: it is defined as cases in which no definitive cause of death was identified.

*Severe pre-eclampsia*: it is defined as an elevation of the blood pressure ≥ 140/90 mmHg after 20 weeks of gestation in a previously normotensive woman with signs of end-organ damage.

*Inadequate antenatal care (ANC)*: it is defined when the total number of contacts are less than eight visits.

*Inclusion/exclusion criteria*: pregnant women attending in labor and who were willing to participate in the study and had stillbirths after 24 weeks of gestation. Those who were not willing and below 24 weeks of gestation were excluded from the study.

*Diagnosis*: the cause of death was established by the attending pediatrician as proposed by the hospital protocols.

Sample size estimation

The sample size of 285 patients with stillbirths was calculated based on the previous prevalence (20%). This sample was calculated to detect a difference of 5% at α = 0.05 with a power of 80%. With the assumption that 10% of the patients might not respond.

Questionnaire

A structured questionnaire designed by the researcher was used to collect data from each subject who was willing to participate in the study. Data were collected by a trained senior registrar in obstetrics and gynecology. The questionnaire included two parts. The first part covered sociodemographic data (maternal age, parity, education, residence, etc.), and the second part covered causes of stillbirths.

Statistics analysis

The Statistical Package for Social Sciences (SPSS) version 24 (IBM Corp., Armonk, NY) was used to analyze data. Data were demonstrated as tables for the descriptive statistics (frequency tables).

Ethical considerations

Written consent was obtained from each participant in the study. The study was reviewed and approved by Sudan Medical Specialization Board (SMSB) and revised by the concerned Hospital Ethics Committee.

## Results

A total of 17,389 deliveries were performed during the study period, of whom 285 were diagnosed with stillbirth. Thus, the incidence of stillbirths was 16/1000. The majority of women were within the age group 25-34 years, 118 (41.4%), followed by the age group 14-24 years 83 (29.1%), then, age group, 35-44 years 76 (26.6%), age group, 35-44 years, 75 (26.6%), and the least was age group 8 (2.9%) 45 years and above.

 Most of the women were from the urban area 93% (n=265), while 7% (n=20) were from the rural areas. Most of the study group received a secondary level of education 96 (33.7%), followed by those with primary or noneducation who accounted for 133 (56.7%), and university and above were 56 (19.6%). Of all women, 221 (77.5%) were nonemployed, while employed women accounted for 64 (22.5%). Out of all cases, multiparous women constituted 101 (35.4%) of cases, followed by primiparous 88 (30.9%), and grand multiparity 96 (33.5%).

 Most of the cases 251(88.1%) in advanced gestational age ≥ 28 weeks gestations, of these, 133 (46.7%) reported between 30 and 35 weeks of gestations, followed by gestational age between 36 and 41 weeks 118 (41.4%) and those presented as early stillbirths < 28 were 34 (11.9%). Singleton pregnancies accounted for 262 (91.9%) of cases, and the remaining were multiple gestations which accounted for 23 (8.1%) cases. Adequate ANC was achieved in 159 (55.8%) cases (Table 1). 

**Table 1:** Frequencies of the sociodemographic variable of the study population. ANC, antenatal care

Variable	Frequency	Percentage (%)
Age		
14-24	83	29.1
25-34	118	41.4
35 – 44	76	26.6
45 years and above	8	2.9
Residence		
Urban	265	93
Rural	20	7
Education		
Illiterates	65	22.8
Primary	68	23.9
University/above	56	19.6
Occupation		
Housewives	221	77.5
Employees	64	22.5
Parity		
Primiparous	88	30.9
Multiparous	101	35.4
Grand multiparous	96	33.7
Gestational age at presentation		
First and second trimester ≤ 26 weeks	34	11.9
Third trimester > 26 weeks	251	88.1
Singleton	262	91.9
Multiple gestations	23	8.1
ANC		
Inadequate ANC	159	55.8
Adequate ANC	126	44.2

 The most common causes of stillbirth were unexplained in 57 (20%) of cases, followed by pre-eclampsia 53 (18.6%), congenital abnormalities 43 (15.1%), and abruption placentae 41 (14.4%). The least common causes were eclampsia 13 (4.6%), ruptured uterus 12 (4.2%), twin-twin transfusion 12 (4.2%), cord prolapse 10 (3.5%), uncontrolled diabetes mellitus (DM) 10 (3.5%), mal presentation 8 (2.6%), gestational DM 7 (2.5%), anemia 7 (2.5%), sepsis 6 (2.1%), antepartum hemorrhage 4 (1.4%), renal disease 1 (0.4%), and toxoplasmosis 1 (0.4%). 

Of all cases, there were 164 (57.5%) macerated stillbirths while fresh stillbirth accounted for 121 (42.5%). Examination of the umbilical cord demonstrated that 124 (42.5%) stillbirths had a short umbilical cord (<35 cm), and the remaining cases had normal cord length (55-60 cm long) (Table 2).

**Table 2:** Causes of stillbirths among the study population. DM, diabetes mellitus

Variable	Frequency	Percentage (%)
Unexplained	57	20.0
Pre-eclampsia	53	14.4
Congenital abnormality	43	15.1
Abruption placentae	41	14.4
Eclampsia	13	4.6
Rupture uterus	12	4.2
Twin-Twin transfusion syndrome	12	4.2
Card prolapse	10	3.5
Uncontrolled DM	10	3.5
Malpresentation	08	2.6
Gestational DM	07	2.5
Anemia	07	2.5
Sepsis	06	2.1
Antepartum hemorrhage	04	1.4
Renal disease	01	0.4
Toxoplasmosis	01	0.4
Macerated stillbirths	164	57.5
Umbilical cord abnormality	50	17.5
Total	285	100

The majority of stillbirths had their weight less than 2.5 kg, 183 (64.2%), followed by weight ranged between 2.5 and 3.9 kg, 99 (34.7%), and weight above 4 kg, 3 (1.1%).

## Discussion

 The present study showed that incidence of stillbirth was 16/1000. Unexplained causes accounted for 20% (n=57) of cases, followed by pre-eclampsia 18.6% (n=53), congenital abnormalities 15.1% (n=43), and abruption placentae 14.4% (n=41). In addition, the less common causes were eclampsia 4.6% (n=13), ruptured uterus 4.2% (n=12), twin-twin transfusion syndrome 4.2% (n=12), cord prolapse 3.5% (n=160), uncontrolled DM 3.5% (n=10), malpresentation 2.6% (n=8), gestational diabetes mellitus 2.5% (n=7), anemia 2.5% (n=7), sepsis 2.1% (n=6), placenta previa 1.4% (n=4), renal disease 0.4% (n=1), and toxoplasmosis 0.4% (n=1).

The incidence of stillbirths varies from 3.4/1000 total births in high-income countries to as high as 36/1000 in the Sub-Saharan and Southern Asia regions [11]. A higher incidence was reported from India, ranging between 24.4% and 41.9% [12]. In Italy, a 10-year retrospective study demonstrated an incidence rate of 2.8%. The latter study also showed a decline in the incidence of intrauterine fetal demise (IUDF) from 3.1‰ in 2005-2008 to 2.3‰ in the period 2013-2015 [9]. Our rate is comparable to Sub-Saharan and some Asian regions but it is definitively higher than that of developed countries. The wide disparity in the incidence rates can be explained by the lack of agreement in the cut-off point of gestational age to define stillbirths, resulting in underestimating the extent of the problem. Furthermore, data on births and death rates are usually under-reported [13]. In this study, our high incidence rate is because our center is the only tertiary center for high-risk pregnancies in the district. 

The high incidence of stillbirths in developing countries is due to preventable causes, which can be reduced by timely decision-making, and an up-to-date protocol for high-risk pregnancies. Also, it is found that infection control, improvement of health facilities, and emergency obstetric care, especially in rural areas are of paramount importance in reducing fetal death [5-6]. 

In this study, the highest percentage of women, 118(41.4%) with stillbirths were between 25 and 34 years of age. This finding contradicts the results obtained by Momo et al. [7], who identified that the older age > 40 years was an independent risk factor for stillbirths. However, our results agreed with Jamal [14], who found that younger age was associated with a significant increase in the incidence of stillbirths. The existing evidence showed that the younger age groups and primigravidae are more likely to develop pre-eclampsia as we demonstrated that 41.4% of stillbirths were within the age group 25-34 years. 

 Unexplained causes remain the leading cause of stillbirths, and it accounts for 25%-50% of all-cause [15]. In the present study, 20% of cases had no identifiable causes, which is consistent with the later study. However, in low-income countries, like in our setting, the unexplained causes may be overestimated because fetal deaths are not subjected to post-mortem study and perinatal pathology as in our setting, due to cultural and religious beliefs.

 Excluding unexplained causes, about 80% of causes in the present study were avoidable including severe pre-eclampsia, which was the most frequent cause that accounted for 18.6%; this is consistent with data from the developing countries that showed a similar incidence rate [16]. In developed countries, the incidence of stillbirths related to severe pre-eclampsia is low as 10.8 per 1000 births [17]. Another population-based study showed a comparable fetal death rate from severe pre-eclampsia (9.2%) [18].

There is a significant decrease in the incidence of stillbirths in high-income countries over the last decades [9, 19]. However, most of this decline has occurred in pregnancies with advanced gestational age, mainly due to implemented health programs targeting this group at this gestational age. In contrast, the high incidence rate of stillbirths in low-income countries is due to a lack of medical resources and a lack of timely decisions on emergency cases. Also, the study showed that conditions occurring in association with pre-eclampsia, such as placental abruption and eclampsia, accounted for 14.4% and 4.6%, respectively.

Placental abruption complicates approximately 1% of all pregnancies. The incidence of placental abruption is reported between 20% and 40%. It has been found that about 44% of placental abruption is associated with maternal hypertension [20]. An Indian study found that pre-eclampsia and its consequences (abruption and eclampsia) accounted for 48.1% of all causes of stillbirths [15], which is greater than what we reported in the present study (37.6%). Our lower rate contradicts the fact that the hypertension rate is more prevalent among African men and women, which can be explained by the study nature and sample size variation. 

The causes of stillbirth are changing in recent years, due to advances in perinatal diagnosis, especially in the developed world. A study from Japan found that the leading cause was fetal malformations which accounted for 43.2% of cases of stillbirths [21], while in developing countries, avoidable causes remain the leading cause. In the present study, the incidence of malformations was 15.1%, comparable to the incidence rate from India (11%) [22]. Congenital malformation in developing countries is determined by gross fetal abnormality without performing postmortem study; therefore, many cases of fetal malformations with minimal abnormalities can be missed. Further, postmortem study is essential in determining the cause of fetal loss and the course of future pregnancies.

In twin-twin transfusion syndrome, if one twin dies, there is a 40% risk of some form of brain injury for the surviving one. Unfortunately, without treatment, approximately almost 80% of twins with twin-twin transfusion syndrome (TTTS) will die [23]. The present study showed that twin-twin transfusion syndrome and cord accident accounted for 4.2% and 3.5%, respectively. In our setting, TTTS is not offered any form of intrauterine surgical intervention.

Diabetes mellitus affects placental blood flow and fetal oxygenation, resulting in fetal demise either direct or mediated through pre-eclampsia and vasculopathy. It has been estimated that the perinatal mortality rate among diabetic mothers is 6.02% [24], which is comparable to 5.1% in our study. 

In the current practice, the role of infection as a cause of stillbirths is minimal, and it has been reported in 2.3% of cases [25], which is comparable to a 2.1% incidence in the present study. In this study, all infections complicate premature rupture membranes.

Neonatal complications in placenta previa causes of fetal death include prematurity, fetal anomalies, and respiratory distress syndrome. In a large study that included 92,983 deliveries, the perinatal mortality rate associated with placenta previa was 2.30% [26-27]. In our study, the rate was 1.4%, which is less than the later study.

Macerated stillbirth indicates inadequate ANC care. We found that 57.5% were stillbirths and the remaining cases were fresh stillbirths, which indicated substandard levels of antenatal and intrapartum care; this can be explained by 55.8% of inadequate care in this study. The short umbilical cord is associated with congenital malformations, intrapartum distress, fetal growth restriction, and a two-fold risk of death [28]. The present study showed 17.5% of cases had a short umbilical cord, which is higher than 10.7% reported in a previous study [29].

The shortcomings of this study are the small sample size, gathering of data from a single-center rather than multiple centers (the latter of which could be more representative of the general population), and the lack of postmortem study which will add further insight on the unseen causes of deaths and planning and counseling couples on the course of future pregnancies.

## Conclusions

The incidence of stillbirth in the present study was 16/1000. Barring the unexplained fetal death, we found that 80% of cases are due to avoidable factors such as pre-eclampsia, abruption, diabetes, and anemia. The relatively high incidence may be partially solved by effective antenatal care programs in rural areas. Also, an intrapartum care protocol is highly needed to reduce the high occurrence of fresh stillbirth (FSB) babies seen in this study (42.5%). Moreover, the lack of perinatal pathology in our setting added another constraint to exact causes of fetal death and greater insight into the planning of future pregnancies.
